# T1 mapping of the liver and the spleen in patients with liver fibrosis—does normalization to the blood pool increase the predictive value?

**DOI:** 10.1007/s00330-020-07447-8

**Published:** 2020-12-11

**Authors:** Verena Carola Obmann, Annalisa Berzigotti, Damiano Catucci, Lukas Ebner, Christoph Gräni, Johannes Thomas Heverhagen, Andreas Christe, Adrian Thomas Huber

**Affiliations:** 1 Department of Diagnostic, Interventional and Pediatric Radiology, Inselspital, Bern University Hospital, University of Bern, Freiburgstrasse, 3010 Bern, Switzerland; 2 Hepatology, Department of Visceral Surgery and Medicine, Inselspital, Bern University Hospital, University of Bern, Bern, Switzerland; 3 Department of Cardiology, Inselspital, Bern University Hospital, University of Bern, Bern, Switzerland

**Keywords:** Magnetic resonance imaging, Elasticity imaging techniques, Liver fibrosis, Cirrhosis, Extracellular space

## Abstract

**Purpose:**

To analyze whether the T1 relaxation time of the liver is a good predictor of significant liver fibrosis and whether normalization to the blood pool improves the predictive value.

**Methods:**

This prospective study was conducted between 03/2016 and 02/2018. One hundred seventy-three patients underwent multiparametric liver MRI at 3 T. The T1 relaxation time was measured in the liver and the spleen, in the aorta, the portal vein, and the inferior vena cava (IVC). T1 relaxation times with and without normalization to the blood pool were compared between patients with (*n* = 26) and without (*n* = 141) significant liver fibrosis, based on a cutoff value of 3.5 kPa in MRE as the noninvasive reference standard. For statistics, Student’s *t* test, receiver operating characteristic (ROC) curve analysis, and Pearson’s correlation were used.

**Results:**

The T1 relaxation time of the liver was significantly longer in patients with liver fibrosis, both with and without blood pool normalization (*p* < 0.001). T1 relaxation time of the liver allowed prediction of significant liver fibrosis (AUC = 0.88), while normalization to the IVC resulted in a slightly lower performance (AUC = 0.82). The lowest performance was achieved when the T1 relaxation times of the liver were normalized to the aorta (AUC = 0.66) and to the portal vein (AUC = 0.62). The T1 relaxation time of the spleen detected significant liver fibrosis with an AUC of 0.68, and 0.51–0.64 with normalization to the blood pool.

**Conclusion:**

The T1 relaxation time of the liver is a good predictor of significant liver fibrosis. However, normalization of the blood pool did not improve the predictive value.

**Key Points:**

*• The T1 relaxation time of the liver is a good predictor of significant liver fibrosis.*

*• Normalization to the blood pool did not improve the predictive value of T1 mapping.*

*• If the blood pool normalization was weighted 30% to the aorta and 70% to the portal vein, the performance was better than normalization to the aorta alone but still lower than normalization to the IVC.*

## Introduction

The prognosis and management of chronic liver disease (CLD) depend strongly on the degree of liver fibrosis in all etiologies [1]. Magnetic resonance elastography (MRE) allows accurate assessment of liver fibrosis with low failure rates and coverage of the whole liver volume [2, 3]. MRE therefore represents the noninvasive reference standard for the assessment of liver fibrosis [4, 5]. One major advantage of MRE is the possibility of combining it with other magnetic resonance imaging (MRI) sequences for liver fat [6, 7] and iron quantification [8] as a one-stop shop. However, MRE equipment is expensive and not yet widely available. A more widely available MRI sequence would therefore be very helpful to detect significant liver fibrosis without the need for expensive additional hardware or time-consuming image post-processing.

One very promising quantitative MRI sequence to do so is T1 mapping. It allows quantification of the T1 relaxation time of the liver and spleen [9, 10] and may be acquired within one breath-hold per slice without the need for any additional hardware or time-consuming image post-processing. Recently, researchers showed that the T1 relaxation time of the liver is significantly longer in patients with liver fibrosis [11, 12]. Other studies demonstrated a longer T1 relaxation time of the spleen in patients with significant portal hypertension [13].

However, T1 is influenced by the patient’s hematocrit [14, 15], blood oxygenation [16], and amount of blood pool [17]. Even if large vessels are excluded from the region of interest (ROI), their value always represents a mix of T1 relaxation times of the liver parenchyma, bile ducts, and blood pool, including afferent arterial and portal vein vessels, liver sinusoids, and efferent liver veins. Since we are mainly interested in the T1 relaxation time of the liver parenchyma, it might be helpful to minimize the effects of the liver blood pool by normalizing it to the inferior vena cava (IVC), to the portal vein, or to the aorta.

The aim of this study was to analyze whether the T1 relaxation time of the liver is a good predictor of significant liver fibrosis and whether normalization to the blood pool improves the predictive value using MRE as a reference standard.

## Method and materials

### Study population

**Fig. 1:**
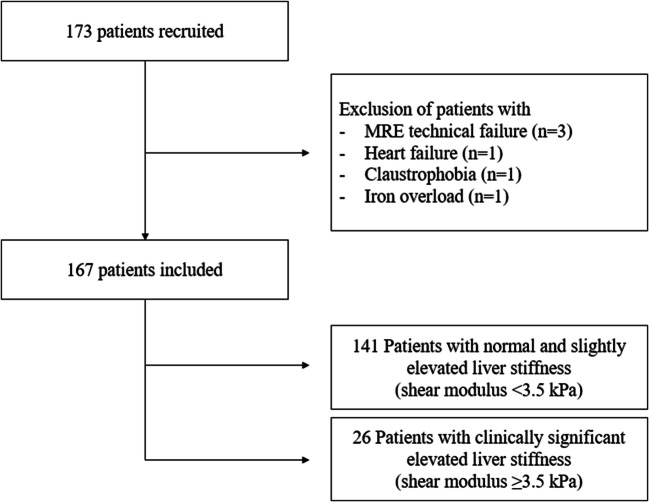
Patient flowchart. A total of 173 patients without prior liver surgery, solid liver lesions, or portal vein thrombosis underwent multiparametric MRI. Six patients have been excluded due to technical failure of MRE (*n* = 3), claustrophobia (*n* = 1), heart failure (*n* = 1), or iron overload (*n* = 1) resulting in an included study population of 167 patients which could be divided into 141 patients with a liver shear modulus < 3.5 kPa and 26 patients ≥ 3.5 kPa). MRE, magnetic resonance elastography

This prospective study was approved by the institutional review board (Kantonale Ethikkommission Bern, IRB number 282-15) and was conducted after obtaining written patient informed consent. All consecutive patients undergoing liver CT and liver MRI with MRE in our institution between 03/2016 and 02/2018 were included in the study. Based on CT images, patients without prior liver surgery, solid liver lesions, or portal vein thrombosis were selected. A total of 173 patients thus underwent multiparametric liver MRI at 3 T, including T1 mapping, proton density fat fraction (PDFF) quantification, and gradient echo–based MRE. Six patients were excluded because of an incomplete MR exam due to claustrophobia (*n* = 1), iron overload (*n* = 1), heart failure (*n* = 1), or technical failure of MRE (*n* = 3) (Fig. 1). There were no further specific inclusion criteria to ensure to cover the entire spectrum from patients without known liver disease to those with no and mild liver fibrosis as well as those patients with advanced liver fibrosis and know cirrhosis. Patients were divided into two groups with and without significantly elevated liver stiffness using a cutoff value of 3.5 kPa in MRE (corresponding to F2 or higher in histology) [18]. A total of 141 patients had a liver shear modulus < 3.5 kPa, while 26 patients had a liver shear modulus ≥ 3.5 kPa. Clinical information and laboratory test results were recorded. Clinical parameters included age, sex, body mass index (BMI), history of diabetes or hypertension, daily drug intake, tobacco use, and alcohol consumption. Biological parameters included dyslipidemia, platelet count, quick value, total bilirubin levels, gamma-glutamyl transpeptidase (GGT), aspartate aminotransferase (AST), alanine aminotransferase (ALT), alkaline phosphatase, albumin, creatinine, and hematocrit.

### MR imaging technique

All liver exams were performed on a 3-T MR system (Verio, Siemens Healthineers) in a fasting state (> 6 h) including T1- and T2-weighted sequences, T1 relaxometry, and MRE. For T1 relaxometry, three single breath-hold (11 ms) axial slices were acquired in the liver using a MOLLI sequence with a 3-3-5 design. ECG was simulated by pulse triggering on the patient’s fingertip. The following parameters were used: repetition time (TR) of 740 ms, echo time (TE) of 1.01 ms, inversion time (TI) of 225 ms (3 inversion pulses, at 65 ms, 145 ms, and 225 ms), and flip angle (FA) of 35°. The slice thickness was 8 mm, the field-of-view (FOV) was 308 × 384 mm, and the matrix was 154 × 192 pixels. For MRE, a gradient echo–based MRE sequence (WIP package 622 provided by Siemens Healthineers) was used. A pneumatic driver (Resoundant®) was placed on the right upper quadrant of the abdomen, transmitting shear waves by continuous acoustic vibrations with a frequency of 60 Hz. Three single-slice acquisitions with 5-mm slice thicknesses were performed on the same level as T1 maps.

### MR imaging analysis

**Fig. 2:**
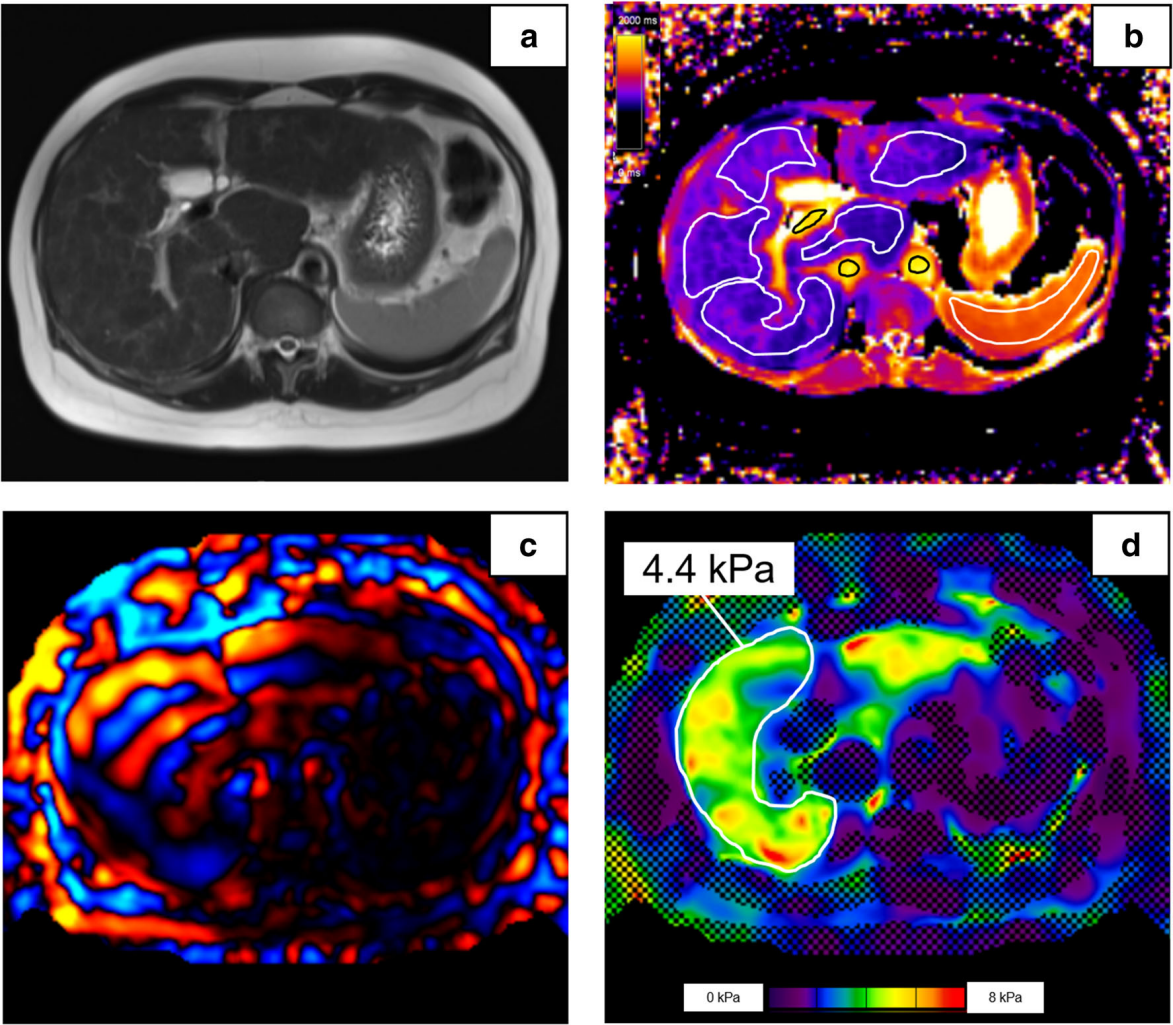
Patient example. Images from a 47-year-old female patient with chronic hepatitis C and liver fibrosis F3 in histology. T2w HASTE sequence (**a**) is shown for anatomical orientation on the same slice as the T1 mapping (**b**) and MRE wave image (**c**) and stiffness map (**d**). ROIs on the T1 map are drawn in the liver (mean 947 ms), spleen (1462 ms), portal vein (1781 ms), IVC (1899 ms), and aorta (1972 ms). The liver T1 relaxation time (mean 947 ms) and stiffness (4.4 kPa) are both elevated. HASTE, half-Fourier single-shot turbo spin-echo; MRE, magnetic resonance elastography; ROI, region of interest; IVC, inferior vena cava

For imaging analysis, regions of interest (ROIs) were drawn in the right liver lobe by an experienced radiologist (V.O., 8 years of experience in hepatic imaging) on T1 maps, as well as on MRE 95% confidence stiffness maps. ROIs on T1 maps were drawn in every liver segment that was visualized in good image quality with a minimal distance of 5 mm to the liver border and to large blood vessels to avoid partial volume effects, with a mean ROI size of 209 pixels. Measurements from all liver segments were then averaged in every patient. Liver areas adjacent to the lung were avoided to exclude air susceptibility effects (10). The T1 relaxation time was measured in the right lobe of the liver and in the spleen, as well as in the aorta, in the portal vein, and in the inferior vena cava (IVC). All vessel measurements were made on the same level of the liver hilus on the level of the portal vein bifurcation. Liver stiffness was measured in the right liver lobe only (Fig. 2). ROIs on MRE stiffness maps were drawn in the liver in the 95% confidence region, which is shown without crosshairs on the map, with a ROI size of 4382 ± 2234 pixels. Measurements from three slices were then averaged to generate the patients’ liver stiffness value. A shear modulus ≥ 3.5 kPa was defined as significant liver fibrosis (corresponding to histology fibrosis stage ≥ F2) [3, 19, 20].

### Statistical analysis

Analysis was performed with the statistical software package R (version 3.4.1, R Foundation for Statistical Computing) [21] and GraphPad Prism (version 7.1, GraphPad Software Inc.). Clinical characteristics were compared between groups using the Wilcoxon test for continuous variables or Fisher’s exact test for categorical variables. The level of significance was *p* < 0.05. MRE liver stiffness was compared with T1 relaxation times alone, as well as T1 relaxation times normalized to the blood pool in the IVC, in the portal vein, in the aorta, and in the spleen. For normalization to the blood pool, T1 relaxation times of the liver were divided by the T1 relaxation times of the blood pool. Since the liver has a dual arterial and venous blood supply, T1 relaxation times were also normalized to the blood pool weighted 30% for the aorta and 70% for the portal vein. To investigate the usefulness of different T1 relaxometry values to predict significant liver fibrosis, Pearson correlation, Student’s *t* test, and receiver operating characteristic (ROC) curve analysis were used. Cutoff values were calculated based on the Youden index. For the purpose of assessing the interrater reliability of T1 measurements, T1 relaxation times of the liver, aorta, portal vein, IVC, and spleen were remeasured by a second reader (D.C., 1 year of experience with hepatic imaging) in twenty patients: ten patients who were randomly selected with a shear modulus < 3.5 kPa and 10 patients who were randomly selected with a shear modulus ≥ 3.5 kPa. The intraclass correlation coefficient (ICC) was then calculated, and ICC classifications of 0.4–0.59 were considered fair, 0.6–0.74 were considered good, and 0.75–1.00 were considered excellent [22].

## Results

### Patient characteristics

The patient characteristics are shown in Table 1. Patients with elevated liver stiffness (shear modulus ≥ 3.5 kPa) showed higher frequencies of daily tobacco and alcohol consumption, higher liver enzymes (AST: 46 ± 22 vs. 28 ± 18, *p* value < 0.004 and GGT: 140 ± 91 vs. 39 ± 40, *p* value < 0.001), and bilirubin (22 ± 18 vs. 9 ± 6, *p* value 0.004), as well as lower thrombocytes (158 ± 100 vs. 245 ± 86, *p* = 0.006) and Quick value (77 ± 17 vs. 96 ± 12, *p* value < 0.001), compared to those of patients with a liver shear modulus < 3.5 kPa. There was no significant difference in BMI between the groups (29 ± 7 vs. 26 ± 7, *p* = 0.12).

**Table 1:** Patient characteristics

	Liver shear modulus < 3.5 kPa	*n*	Liver shear modulus ≥ 3.5 kPa	*n*	*p* value
Age, years	50 ± 15	141	59 ± 9	26	< 0.001
Male, %	72 (51%)	141	15 (58%)	26	0.404
Shear modulus, kPa	2.23 ± 0.49	141	6.01 ± 3.34	26	< 0.001**
Tobacco, *n* (%)	25 (18%)	138	15 (60%)	25	< 0.001**
Daily alcohol consumption, *n* (%)	13 (9%)	140	17 (68%)	25	< 0.001**
Diabetes, *n* (%)	10 (10%)	96	7 (30%)	23	0.005*
Hypertension, *n* (%)	29 (21%)	138	12 (48%)	25	0.016
BMI, kg/m^2^	26 ± 7	137	29 ± 7	22	0.120
PDFF, %	11 ± 8	141	13 ± 11	26	0.427
AST, U/l	28 ± 18	56	46 ± 22	20	< 0.004*
ALT, U/l	39 ± 50	74	39 ± 23	20	0.965
GGT, U/l	39 ± 40	65	140 ± 91	21	< 0.001**
Alkaline phosphatase, U/l	74 ± 30	57	102 ± 48	19	0.021*
Bilirubin, μmol/l	9 ± 6	51	22 ± 18	21	0.004*
Albumin	35 ± 7	43	34 ± 7	21	0.224
Thrombocytes	245 ± 86	35	158 ± 100	16	0.006*
Quick, %	96 ± 12	64	77 ± 17	21	< 0.001**
APRI	0.4 ± 1.1	34	1.7 ± 2.0	15	0.032*
Creatinine, μmol/l	79 ± 20	93	80 ± 23	23	0.914

Values are the mean ± SD or *n*. *p* values were calculated using the Mann-Whitney *U* or Fisher’s exact test, as appropriate. Comparisons between the two patient groups are indicated with * if *p* < 0.05 and ** if *p* < 0.001

*BMI*, body mass index; *PDFF*, proton density fat fraction; *AST*, aspartate aminotransferase; *ALT*, alanine aminotransferase; *GGT*, gamma-glutamyl transferase; *APRI*, aspartate aminotransferase-to-platelet ratio index

According to MRE as the reference standard, 103 patients had a liver shear modulus of less than 2.5 kPa, corresponding to normal liver stiffness; 23 patients had a liver shear modulus of 2.5 to ≤ 2.9, corresponding to normal or inflammation; and 15 patients had a liver shear modulus 2.9 to ≤ 3.5 kPa (F1–2), 5 patients 3.5 to ≤ 4.0 kPa (F2–3), 8 patients 4.0 to ≤ 5.0 kPa (F3–4), and 13 patients more than 5.0 kPa (F4) using cutoffs as described by Srinivasa et al [3].

Of the 26 patients with elevated liver stiffness (shear modulus ≥ 3.5 kPa), 23 had known liver fibrosis (1 patient with a histology fibrosis stage F1, 2 patients with F2, 6 patients with F3, and 14 patients with F4 in histology or clinically established diagnosis of liver cirrhosis), while chronic liver disease was not known in 3 patients who were lost to follow-up (1 patient with metabolic syndrome and 2 patients with cancer without liver metastasis). The etiology of liver disease in patients with elevated liver stiffness was viral hepatitis (*n* = 9), alcohol-induced liver disease (*n* = 10), NAFLD/NASH (*n* = 3), and cryptogenic liver cirrhosis (F4) in histology in 1 patient and unknown in the 3 patients who were lost to follow-up, as described above (Table 2).

**Table 2:** Etiology of chronic liver disease in the study population

Etiology	Liver shear modulus < 3.5 kPa (*n* = 141)	Liver shear modulus ≥ 3.5 kPa (*n* = 26)
Viral hepatitis	*N* = 5 (without known fibrosis)	*N* = 9
ALD	*N* = 0	*N* = 10
NAFLD/NASH	*N* = 4 (1 F0; 2 F1; 1 F2)	*N* = 3
Cryptogenic cirrhosis	*N* = 0	*N* = 1
Unknown reason/lost for FU	*N* = 0	*N* = 3
No known liver disease	*N* = 132	*N* = 0

In 26 patients with a liver shear modulus ≥ 3.5 kPa (corresponding to a liver fibrosis stage f2 or higher), most prevalent etiology of chronic liver disease was viral hepatitis (*n* = 9) and ALD (*n* = 10). Of 141 patients with a liver shear modulus < 3.5 kPa, 132 had no known chronic liver disease. The remaining 9 patients with chronic liver disease had viral hepatitis without fibrosis (*n* = 5) or NAFLD/NASH without significant fibrosis (*n* = 4)

*ALD*, alcoholic liver disease; *NAFLD/NASH*, non-alcoholic fatty liver disease/non-alcoholic steatohepatitis; *FU*, follow-up

Of the 141 patients without elevated liver stiffness (shear modulus < 3.5 kPa), 4 had liver biopsy with a diagnosis of NAFLD/NASH (1 patient with a histology fibrosis stage F2, 2 patients with F1, and 1 patient with F0), 5 patients had chronic viral hepatitis without known liver fibrosis, while the remaining 132 patients had no known chronic liver disease. In patients with significantly elevated liver stiffness (shear modulus ≥ 3.5 kPa, corresponding to a liver fibrosis stage f2 or higher), 19/26 patients (73%) had liver steatosis (PDFF ≥ 5%), while 10/26 patients (38%) had moderate to severe liver steatosis (PDFF ≥ 15%). In patients without significantly elevated liver stiffness (shear modulus < 3.5 kPa), 102/141 patients (73%) had liver steatosis (PDFF ≥ 5%), while 19/141 patients (13%) had moderate or severe liver steatosis (PDFF ≥ 15%).

### T1 mapping results

**Fig. 3:**
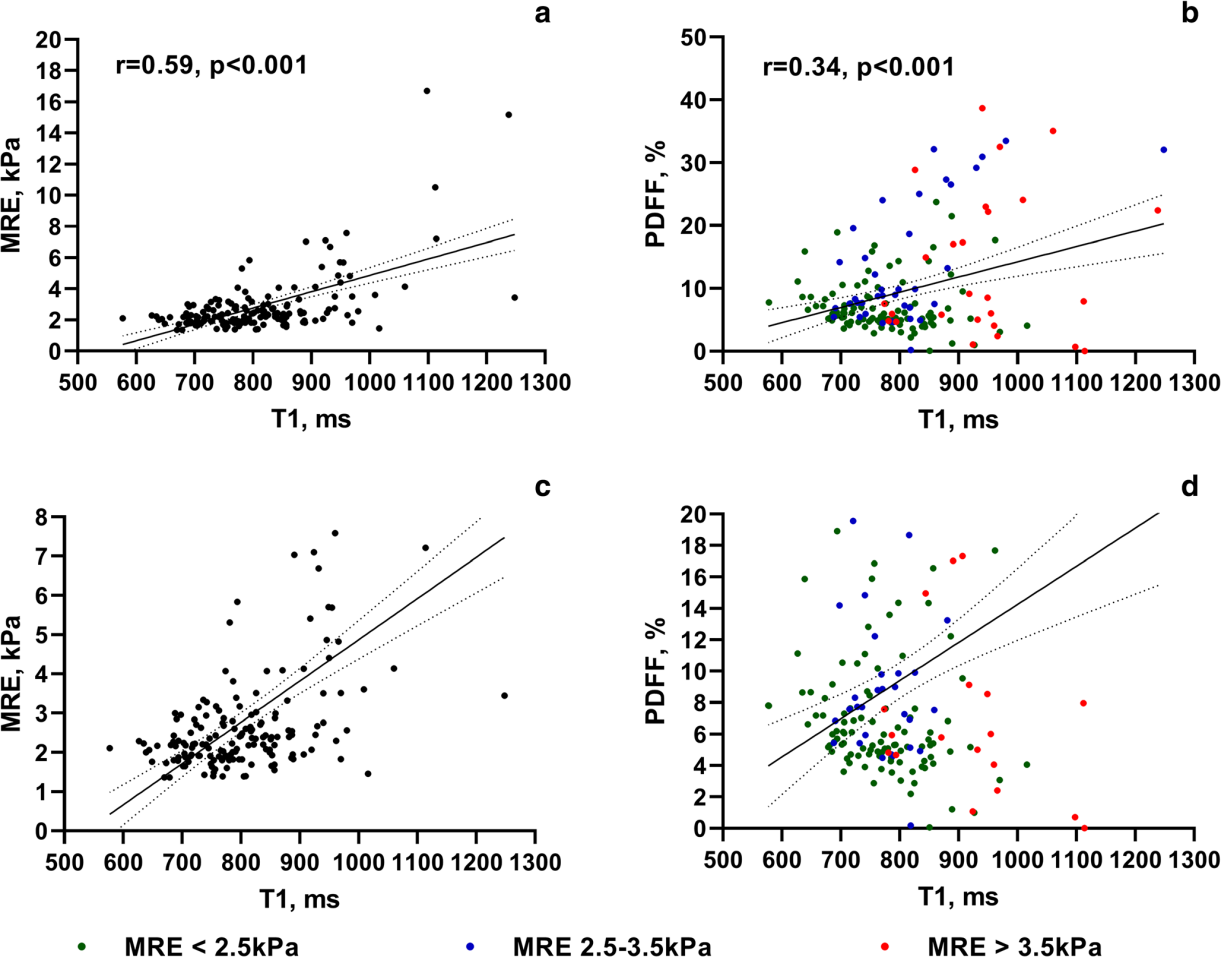
Scatterplots and correlation analysis. Scatterplots illustrate the relationship between MRE, T1, and PDFF in **a** and **b**. For better visualization, **c** and **d** represent enlarged views of a and b. In **b** and **d**, patients were color-coded to reflect the degree of their underlying liver stiffness based on MRE. MRE, magnetic resonance elastography; PDFF, proton density fat fraction

T1 relaxation time in the liver, both with and without blood pool normalization, was significantly longer in patients with a liver shear modulus ≥ 3.5 kPa compared to those with a liver shear modulus < 3.5 kPa (943 ± 114 ms and 781 ± 90 ms, *p* < 0.001). T1 relaxation time in the spleen was significantly longer in patients with elevated liver stiffness as well (1296 ± 130 ms and 1220 ± 109 ms, *p* = 0.011), although the differences were smaller than for the T1 relaxation time of the liver (Table 3). In the Pearson analysis, longer T1 relaxation times of the liver correlated well with higher liver shear modulus (*r* = 0.59, *p* < 0.001), while relaxation times of the liver normalized to the blood correlated less with higher liver shear modulus (*r* = 0.2–0.46, *p* < 0.001–0.06) (Table 4). Although fat is a known confounding factor of T1 relaxation time [23], correlation between T1 relaxation times and PDFF was weak (*r* = 0.34, *p* < 0.001), while correlation of T1 with MRE was much better (*r* = 0.59, *p* < 0.001). Interestingly, the scatterplot shows that patients with higher PDFF also tend to have high liver stiffness as an indicator of coexisting inflammation and/or fibrosis (Fig. 3). The correlation between higher liver stiffness and longer T1 time of the spleen was rather low (*r* = 0.23, *p* = 0.004).

**Table 3:** Comparison of T1 relaxation times in patients with and without significant liver fibrosis

	Liver shear modulus < 3.5 kPa	*n*	Liver shear modulus ≥ 3.5 kPa	*n*	*p* value
T1 liver, ms	781 ± 90	141	943 ± 114	26	< 0.001**
T1 spleen, ms	1221 ± 109	130	1296 ± 130	25	0.003*
T1 aorta, ms	1609 ± 683	133	1628 ± 336	25	0.835
T1 cava, ms	1639 ± 180	140	1645 ± 202	24	0.886
T1 liver/cava	0.48 ± 0.07	140	0.58 ± 0.09	24	< 0.001**
T1 liver/aorta	0.51 ± 0.10	133	0.61 ± 0.17	25	< 0.001**
T1 liver/portal vein	0.50 ± 0.06	82	0.52 ± 0.07	10	0.259
T1 liver/aorta and portal vein (30%/70%)	0.50 ± 0.07	77	0.53 ± 0.07	10	0.130
T1 liver/spleen	0.64 ± 0.07	130	0.73 ± 0.09	25	< 0.001**
T1 spleen/cava	0.75 ± 0.10	129	0.80 ± 0.10	24	0.044*
T1 spleen/aorta	0.80 ± 0.16	122	0.83 ± 0.22	25	0.350
T1 spleen/aorta and cava (50%/50%)	0.78 ± 0.13	123	0.81 ± 0.11	24	0.293
T1 spleen/aorta and cava (30%/70%)	0.78 ± 0.20	123	0.80 ± 0.10	24	0.393

Values are the mean ± SD or *n*. *p* values were calculated using Student’s *t* test

Comparisons between the two patient groups are indicated with * if *p* < 0.05 and ** if *p* < 0.001

**Table 4:** Pearson correlation between T1 values and liver stiffness

	Pearson correlation r	*p* value
T1 liver, ms	0.59	< 0.001**
T1 spleen, ms	0.23	0.004*
T1 liver/cava	0.45	< 0.001**
T1 liver/aorta	0.43	< 0.001**
T1 liver/spleen	0.46	< 0.001**
T1 liver/portal vein	0.20	0.058
T1 liver/aorta and portal vein (30%/70%)	0.36	< 0.001**
T1 liver/spleen	0.46	< 0.001**
T1 spleen/cava	0.11	0.165
T1 spleen/aorta	0.15	0.080

Values represent the Pearson correlation coefficient with the corresponding *p* values. Comparisons between the two patient groups are indicated with * if *p* < 0.05 and ** if *p* < 0.001

### ROC analysis

**Fig. 4:**
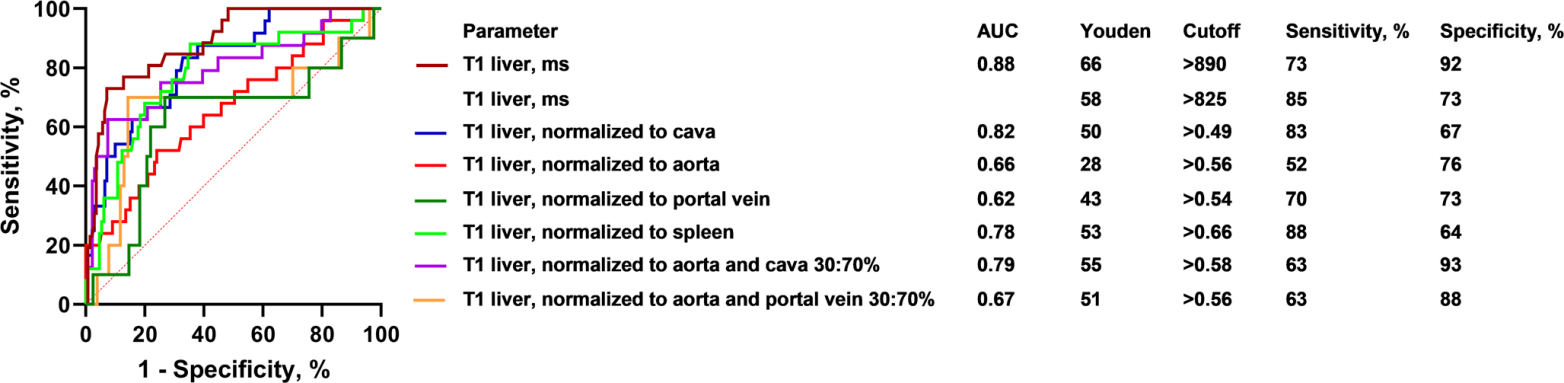
ROC analysis. ROC curves for the T1 mapping results of the liver, as well as those normalized to the cava, aorta, and portal vein, distinguishing between patients with and without elevated liver stiffness (shear modulus < 3.5 kPa vs. ≥ 3.5 kPa), are shown. AUC values as well as the number of measurements are indicated. ROC, receiver operating characteristics; AUC, area under the ROC curve

The ROC analysis (Fig. 4) confirmed the good performance of T1 relaxation times of the liver to separate patients with a liver shear modulus < 3.5 kPa and patients with a liver shear modulus ≥ 3.5 kPa (AUC = 0.88). The different cutoff values based on the Youden index are shown in Fig. 4. For T1 relaxation time of the liver, two cutoff values are shown. The higher cutoff value of > 890 ms is optimized using the Youden index, with a specificity of 92% and a sensitivity of 73% for significant liver fibrosis. The lower cutoff of > 825 ms is optimized for high sensitivity with a specificity of 73% and a specificity 85%. When the T1 relaxation time of the liver was normalized to the blood pool, the best performance was obtained when normalizing to the IVC (AUC = 0.82), while the performance of T1 relaxation times normalized to a mixed arterial and portal venous pool with a weighting of 30%:70% was lower (AUC = 0.67). The lowest performance was achieved when the T1 relaxation times of the liver were normalized to the aorta (AUC = 0.66) and the portal vein (AUC = 0.62). The T1 relaxation times of the spleen allowed a discrimination of the two patient groups with an AUC = 0.68, while normalization of the T1 relaxation times of the spleen to the blood pool showed slightly lower performance (AUC = 0.51–0.64, *p* = 0.03 for IVC and *p* = 0.831 for aorta normalization).

### Robustness of the measurements and interreader reliability

T1 relaxation times of the liver were measured in all 167 patients. While aortic and IVC measurements were possible in 95% and 98%, the portal vein was captured in only 55% of the patients on T1 maps (Table 5). T1 mapping of the spleen was measured and of good quality in 155 patients, not measurable in 5 patients, and 7 patients had a post-splenectomy status. Interobserver reliability within our group was excellent for all measurements (ICC = 0.84–0.97), except for the measurements for the T1 ratio in the liver to the aorta and portal vein (ICC = 0.74), which were slightly lower.

**Table 5:** Robustness of the measurements and intraclass correlation coefficient for T1 relaxation times

	*N* < 3.5 kPa	*N* ≥ 3.5 kPa	*N* total	%	ICC
T1 liver	141	26	167	100	0.97
T1 liver and cava	140	24	164	98	0.92
T1 liver and aorta	133	25	158	95	0.86
T1 liver and portal vein	82	10	92	55	0.91
T1 liver and aorta and portal vein	77	10	87	52	0.74
T1 liver and spleen	130	25	155	93	0.86
T1 spleen	130	25	155	93	0.92
T1 spleen and cava	129	24	153	92	0.91
T1 spleen and aorta	122	25	147	88	0.85

Values are the number of patients in which T1 measurements could be performed with excellent quality. The intraclass correlation coefficient (ICC) between both readers is noted

## Discussion

This is the first study to demonstrate that the T1 relaxation time of the liver is significantly longer in patients with liver fibrosis, both with and without blood pool normalization. Overall, the T1 relaxation time of the liver without blood pool normalization was the best predictor of significant liver fibrosis, based on an MRE cutoff value of 3.5 kPa or higher [24]. With blood pool normalization, better results were observed in the IVC than in the portal vein and in the aorta. Measurements were more difficult in the portal vein than in the IVC and in the aorta due to its horizontal course through the acquired slices [25]. Unlike the IVC and the aorta, the portal vein was not always captured and was prone to partial volume effects.

The blood pool in the vena cava is probably more similar to the blood pool in the liver sinusoids, than the blood pool in the aorta. This might explain why the normalization of T1 relaxation times to the vena cava showed better results than normalization to the aorta. Interestingly, normalization to a combined mean value of the aorta and portal vein resulted in better results with a higher specificity than normalization to the aorta or the portal vein alone. This indicates that blood pool normalization works better, the closer we get to the T1 relaxation times of the liver sinusoids. Another important confounder of normalization to the aorta and to the portal vein might be the different afferent vascularization of the liver, with an arterial portion of approximately 30% in a normal liver, which increases significantly in liver fibrosis [26], as well as flow phenomenon in the aorta influencing the measured T1 relaxation time. Whether adapting the arterial portion in relation to the degree of fibrosis or angled, orthogonal acquisitions of T1 maps to the portal vein might increase the predictive value should be investigated in a subsequent study.

Our results are in line with the findings of other groups, who have shown that liver T1 relaxation time is a good predictor of fibrosis [11, 12, 27, 28] and may even predict clinical outcome [29]. Yoon et al published T1 relaxation times of the liver between 879 and 1042 ms in patients with liver cirrhosis and different Child-Pugh scores [30], which is in the same range as measured in our patients. Other studies investigated T1 relaxation time of the liver normalized to the skeletal muscles [31]. However, to our knowledge, this is the first study investigating the T1 relaxation time of the liver normalized to the blood pool in the IVC, in the aorta, and in the portal vein.

The T1 relaxation time of the spleen was significantly longer in patients with liver fibrosis, but the difference between patients with and without liver fibrosis was much smaller in the spleen than in the liver. This observation was confirmed by ROC analysis. A probable explanation for the higher T1 relaxation time of the spleen in patients with significant liver fibrosis is portal hypertension with subsequent splenic congestion. For the detection of significant fibrosis using splenic parameters, splenic volumetry has an AUC of 0.83 [32], and the 2D diameter of the interpole distance has an AUC of 0.88 [33], both of which are higher than splenic T1 mapping (AUC 0.68). Levick et al showed that a longer T1 relaxation time of the spleen correlated with an increasing hepatic venous pressure gradient (HVPG) [13]. However, this might be masked by Gamna-Gandy bodies in the spleen in patients with portal hypertension, which would shorten the T1 relaxation time [34]. The same phenomenon may be observed when measuring significantly increased splenic stiffness in patients with increasing degrees of liver fibrosis [35, 36]. Another study from Reiter et al has demonstrated a correlation between splenic stiffness and the degree of liver fibrosis in multifrequency tomoelastography. The combination of hepatic and splenic shear wave speed resulted in an even higher prediction for liver fibrosis, while the best prediction was achieved with 60 Hz [37].

T1 relaxation time measurements of the liver and spleen were very robust with excellent interreader reliability. When normalizing the blood pool, measurements in the portal vein were less robust and reproducible. Unlike the aorta and the IVC, the portal vein was often not covered on the acquired slices or showed partial volume effects due to its rather horizontal course through the axial slices. To ensure high reproducibility of the T1 relaxation time measurements of the liver, the ROI should not be drawn within a distance of 5 mm to the liver border and to large blood vessels to avoid partial volume effects. Liver areas adjacent to the lung should be avoided because of partial volume and susceptibility effects [10]. Nevertheless, even if those criteria are taken into account, it is inevitable that smaller vessels and the blood pool in the liver sinusoids are included in any liver ROI. The measured T1 relaxation time therefore represents a mix of tissue composition, bile ducts, and blood pool, including arteries, portal veins, liver veins, and liver sinusoids.

As demonstrated in this study, the liver T1 relaxation time is more influenced by the venous blood pool in the liver sinusoids than the blood in the dual afferent arterial and portal venous vascular system. This might be of interest when T1 mapping with extracellular contrast medium is used to calculate extracellular volume (ECV) fraction [38]. The ECV is calculated as the difference of relaxation rates (R1 = 1/T1) of the blood and liver parenchyma before and after intravenous administration of an extracellular contrast medium, corrected for the hematocrit. The initial results have shown that the ECV fraction increases with the degree of liver fibrosis [39]. Based on these findings, ECV calculation in the liver might be more accurate when measured in the IVC and not in the aorta. This makes sense since the blood hematocrit, used to calculate ECV, is normally measured in a venous blood sample. A comparison of different ECV values of the liver comparing blood pool measurement in the aorta and in the IVC should be investigated in upcoming studies.

There are several other confounders of T1 relaxation time in the liver, including steatosis, inflammation, and portal hypertension. Both inflammation and portal hypertension are influencing liver stiffness in MRE as well, so a differentiation is not possible with the current study design and should be subject to further investigations. A higher degree of liver steatosis, as measured with PDFF in this study, correlated with a longer T1 relaxation time. However, patients with a high degree of liver steatosis also showed higher degrees of liver stiffness in MRE. This indicates coexistence of inflammation and/or fibrosis in patients with significant liver steatosis.

There are several limitations of our study. First, we did not perform liver biopsy in most patients because a large portion of our patients did not have significant liver fibrosis. Hence, our study population mirrors a daily routine patient spectrum in a radiology department. MRE has been proven to reflect the degree of liver fibrosis very accurately compared to histology [2]. In addition, it covers the whole liver volume and not just a 1/40,000 volume of the liver with potential sampling errors in histology (38, 39). Another limitation is the relatively small proportion of patients with significantly elevated liver stiffness compared to the bigger proportion of patients without known liver disease and thus normal liver stiffness measurements. Again, this reflects daily routine in a radiology department where we intend to screen for liver fibrosis in a large population, including patients without known liver fibrosis. However, this skewed patient distribution might have resulted in a consecutive overestimation of performance of T1 mapping of the liver itself while the main question of the study, the impact of normalization to the blood pool, should not be substantially impacted.

## Conclusion

The T1 relaxation time of the liver is a good predictor of significant liver fibrosis. However, normalization to the blood pool did not improve the predictive value.

## Abbreviations

ALT: Alanine aminotransferase
AST: Aspartate aminotransferase
AUC: Area under the curve
BMI: Body mass index
CLD: Chronic liver disease
GGT: Gamma-glutamyl-transpeptidase
HVPG: Hepatic venous pressure gradient
IVC: Inferior vena cava
MOLLI: Modified Look-Locker inversion recovery sequence
MRE: Magnetic resonance elastography
MRI: Magnetic resonance imaging
PDFF: Proton density fat fraction
ROC: Receiver operating characteristics
ROI: Region of interest
